# Socioeconomic Differentials in the Immediate Mortality Effects of the National Irish Smoking Ban

**DOI:** 10.1371/journal.pone.0098617

**Published:** 2014-06-02

**Authors:** Sericea Stallings-Smith, Pat Goodman, Zubair Kabir, Luke Clancy, Ariana Zeka

**Affiliations:** 1 Institute for the Environment, Brunel University, London, United Kingdom; 2 Environmental Health Sciences Institute, Dublin Institute of Technology, Dublin, Ireland; 3 TobaccoFree Research Institute Ireland, Dublin, Ireland; 4 Department of Epidemiology and Public Health, University College Cork, Cork, Ireland; Universität Bochum, Germany

## Abstract

**Background:**

Consistent evidence has demonstrated that smoking ban policies save lives, but impacts on health inequalities are uncertain as few studies have assessed post-ban effects by socioeconomic status (SES) and findings have been inconsistent. The aim of this study was to assess the effects of the national Irish smoking ban on ischemic heart disease (IHD), stroke, and chronic obstructive pulmonary disease (COPD) mortality by discrete and composite SES indicators to determine impacts on inequalities.

**Methods:**

Census data were used to assign frequencies of structural and material SES indicators to 34 local authorities across Ireland with a 2000–2010 study period. Discrete indicators were jointly analysed through principal component analysis to generate a composite index, with sensitivity analyses conducted by varying the included indicators. Poisson regression with interrupted time-series analysis was conducted to examine monthly age and gender-standardised mortality rates in the Irish population, ages ≥35 years, stratified by tertiles of SES indicators. All models were adjusted for time trend, season, influenza, and smoking prevalence.

**Results:**

Post-ban mortality reductions by structural SES indicators were concentrated in the most deprived tertile for all causes of death, while reductions by material SES indicators were more equitable across SES tertiles. The composite indices mirrored the results of the discrete indicators, demonstrating that post-ban mortality decreases were either greater or similar in the most deprived when compared to the least deprived for all causes of death.

**Conclusions:**

Overall findings indicated that the national Irish smoking ban reduced inequalities in smoking-related mortality. Due to the higher rates of smoking-related mortality in the most deprived group, even equitable reductions across SES tertiles resulted in decreases in inequalities. The choice of SES indicator was influential in the measurement of effects, underscoring that a differentiated analytical approach aided in understanding the complexities in which structural and material factors influence mortality.

## Introduction

The Republic of Ireland was the first country in the world to implement a national workplace smoking ban on March 29, 2004. The implementation of this comprehensive legislation, including a ban on smoking in restaurants, pubs, and bars, resulted in large immediate decreases in mortality due to ischemic heart disease (IHD), stroke, and chronic obstructive pulmonary disease (COPD) [1]. Previous studies have shown that mortality rates for IHD [2], stroke [3], and COPD [4] are greater in persons of lower socioeconomic status (SES). However, the impact of the national Irish smoking ban on inequalities in mortality is unknown.

A recent study on the global burden of disease demonstrated that tobacco smoking including secondhand smoke was the leading risk factor for death and disability-adjusted life years in North America and Western Europe and the second leading risk factor globally, with a global mortality burden of 6.3 million deaths [5]. Echoing the fundamental research of Geoffrey Rose [6], it was suggested that population-wide public health policies can most effectively save lives by tackling the major risk factors of disease burden, where even small reductions in population exposure can result in considerable health improvements [5]. However, when addressing population-wide risk factors, the impact on inequalities should also be considered. Most inequalities in mortality are attributable to non-communicable diseases, with the highest rates occurring in the most deprived groups; importantly, these inequalities in non-communicable diseases are largely driven by the social gradient in smoking [7]. In Ireland, manual occupation groups and unemployed groups have the greatest prevalence of active smoking in the population [8], [9]. These occupational groups also have greater rates of mortality due to cardiovascular and respiratory diseases [10].

When assessing the effects of a population-wide intervention, such as a smoking ban policy, it is important to consider that health benefits may not be equivalent among population subgroups as other factors will determine variability in risk [6] and impact the existing social patterning of health [11]. Since most risk factors for smoking and smoking-related diseases are modified by SES, it is plausible that the resulting health effects following the implementation of a comprehensive smoking ban policy will be distributed differently across SES groups. Preliminary evidence has indicated that workplace smoking bans may sometimes widen existing inequalities [12], though evidence is limited as few epidemiological studies of smoking ban effects have examined post-ban differentials by SES and findings have been inconsistent [13]–[17]. Of these studies, only two have included mortality events in analyses of an adult population, with respective outcomes of acute coronary events and stroke, and have yielded contradictory findings [15], [16]. Therefore, the impacts of smoking ban policies on inequalities in mortality remain to be elucidated.

Previous research has shown that different indicators and classifications of SES, though generally resulting in consistent associations with health, are not always equivalent measures [18]–[21]. For example, structural SES indicators, such as education or nationality, represent aspects of power, social standing, and the potential for social inclusion; whereas material SES indicators, such as housing tenure or car access, represent the resources available to provide opportunities for a healthy life [22]–[24]. However, the influence of these indicators can change over time and interact through different mechanisms to influence health status and, subsequently, mortality [25]. Therefore, the use of multiple indicators to approximate SES can aid in elucidating how structural and material factors discretely influence associations with health outcomes.

No study has yet examined the influence of discrete SES indicators on the measurement of post-smoking ban mortality effects. This study expands previous work which demonstrated immediate mortality reductions in IHD, stroke, and COPD mortality following implementation of the national Irish smoking ban [1] and includes an extended analysis with mortality data for the years 2008–2010 to examine monthly effects by discrete SES indicators and a composite index.

## Methods

### Data Sources for the Republic of Ireland

National mortality data were obtained from the Central Statistics Office (CSO) Ireland for the study period of 2000–2010. Mortality data were coded according to the *International Classification of Diseases, 9^th^ Revision* (ICD-9) from 2000–2006 and according to the *International Classification of Diseases, 10^th^ Revision* (ICD-10) from 2007–2010. Analyses were conducted for the following smoking-related causes of death: IHD (410–414, 429.2/I20–I25), stroke (430–438/I60–I69), and COPD (490–492, 494–496/J40–J44, J47).

To calculate the age and gender-specific population offset for use in statistical modelling and for information on area-level SES indicators, census data for the years 2002 and 2006 were obtained from the CSO Ireland [26]. To enable adjustment for potential confounding due to epidemics of influenza, weekly influenza-like illness (ILI) surveillance data were obtained from the Irish Health Protection Surveillance Centre for the influenza seasons (October-May) of 2000–2001 to 2010–2011 [27]. ILI activity for the influenza season of 1999–2000 was approximated using published data from the European Influenza Surveillance Scheme [28]. Monthly smoking prevalence data from a nationally representative computer-assisted telephone survey of 1,000 persons per month, ages ≥15 years, were obtained from the Ireland Office of Tobacco Control (OTC) for the months of July 2002-December 2010 [9]. A linear regression fitted to OTC data was used to approximate smoking prevalence for 2000–2001.

### SES Indicators

There are 34 local authorities in Ireland, composed of 29 county councils and five city councils. Based upon previous research [29]–[32] and data availability at the level of local authority area, the following structural SES indicators were selected for analyses: education, occupation, foreign nationality, and family composition, along with three material SES indicators: unemployment, housing tenure, and car access. As income data were not available for every local authority area, housing tenure and car access were used to approximate material resources [33], [34].

The Irish census offered several response groups within each SES indicator. For example, the census question regarding educational status provided 14 response possibilities. As a result, it was necessary to collapse the indicator groupings for further analysis, which in the case of education resulted in three pooled groups of low, intermediate, and high. Since the data in each response group were measured as percentages, Spearman rank order correlation tests were conducted to explore relationships between each group within SES indicators to inform the designation of deprivation boundaries.

Census categories capturing non-response were ≤5% in each local authority area for all SES indicators except education (range: 3–9%). Since the non-response group for educational status was correlated with the no education group, non-response frequencies were combined with no education and primary education in the low education grouping. This was consistent with previous research demonstrating that survey non-response and educational item non-response are associated with socioeconomic disadvantage [35]–[38].

The unskilled, semi-skilled, and skilled manual occupation groups were highly correlated, indicating that the appropriate occupational grouping was in the binary form of manual versus non-manual. The suitability of this grouping is consistent with previous evidence from Ireland demonstrating a distinct difference in smoking prevalence between manual and non-manual occupations, with manual workers being more than twice as likely to smoke daily as their non-manual counterparts [8].

For the other five SES indicators, identifying deprivation boundaries was straightforward as the divisions for the collapsed groupings were intuitively binary. The result was that persons either fell in one group or the other. Specifically, persons could either be Irish/UK nationals or non-Irish/non-UK nationals, with a family composition of ≥5 persons or a family composition of ≤4 persons, employed or unemployed, living in owned housing or rented/free housing, with car access or no car access. Consistent with previous research [31], only the SES indicator groupings representing conditions of deprivation were selected for further analyses.

### Statistical Analyses

Census data for each of the SES indicator groupings from the years 2002 and 2006 were linearly interpolated to determine the remaining values for 2000–2010. Percentages of each SES indicator were then calculated for the 34 local authority areas in Ireland for the full study period. Descriptive analyses were conducted to confirm that each SES indicator had sufficient variability to detect an effect in analyses of the mortality data. Spearman rank order correlation tests were then conducted to explore relationships between each of the SES indicators.

A baseline principal component analysis (PCA) with varimax rotation, the most efficient method for obtaining simple structure [39], was conducted to jointly analyse the seven, discrete SES indicators, all expressed as a percentage: low education, manual occupation, non-Irish/non-UK nationality, ≥5 person families, male unemployment, rented/free housing tenure, and no car access. Based upon the Kaiser-Guttman rule [40], and confirmed by a scree plot [39], two factors were extracted, explaining 81% of the overall variance. The first factor loaded highly on the education, occupation, foreign nationality, and family composition indicators, characterising a structural factor [23], [41]. The second factor loaded highly on the indicators of unemployment, housing tenure, and car access, characterising a material factor [23], [41]. The algebraic sum of these two factors was used as the composite measure of SES for each local authority [29]–[31].

Each of the area-level SES indicators and the composite index were assigned to IHD, stroke, and COPD deaths in the Irish population by local authority area. The analysis was restricted to mortality events in ages ≥35 years to reflect the population at risk for smoking-related mortality. The distributions for the composite SES index and each of the SES indicators across the 34 local authority areas were divided into tertiles, a categorisation also employed in previous social epidemiology research [42], [43]. A narrower categorisation of the SES indices was not possible due to insufficient monthly counts by age and gender for each of the mortality causes.

Poisson regression with interrupted time-series analysis was then conducted to examine monthly age and gender-standardised mortality rates for the period of 2000–2010, stratified by tertiles of each SES indicator and the composite index. Methodological details of the Poisson regression analyses and adjustment for potential confounding factors have been reported elsewhere [1]. Briefly, all models were designated to account for the underlying mortality trend, the step change occurring in the month following smoking ban implementation, and the post-ban annual change in trend, with adjustments for season, influenza, and smoking prevalence in all models. Seasonal adjustments were based upon calendar months with winter defined as December-February, spring as March-May, summer as June-August, and autumn as September-November. Periods of high ILI activity were defined as months in which the reported rate of ILI was ≥60/100,000, roughly twice the background rate of ILIs for the Republic of Ireland. Smoking prevalence adjustments were based upon annual means.

All analyses were conducted using SAS version 9.2, with the FACTOR procedure for PCA [44] and the GLIMMIX procedure for statistical modelling [45]. For the presentation of results, beta coefficients were exponentiated to derive rate ratios (RR).

To test for statistically important differences between effect estimates of SES tertiles, 95% confidence intervals were calculated as: 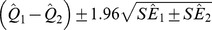
and 90% confidence intervals were calculated as 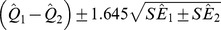, where 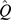 *_1_* and 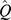 *_2_* were the estimates for two tertiles (for example, the least and most deprived) and *SÊ_1_* and *SÊ_2_* were their respective standard errors [46].

### Sensitivity Analyses

Since education was the only ternary SES indicator and all others were binary, an additional PCA (Sensitivity Analysis 1) was conducted with the inclusion of the high education variable to capture the two tails of the educational distribution, as recommended in previous social research [31]. Additionally, in previous studies wherein a composite SES index was generated from census data, the unemployment indicator was composed of males only [29], [30]. In Ireland, labour force participation is indeed greater for males than that for females [47]. However, from 2001–2007, female labour force participation grew from 48% to 55% [47], demonstrating that females were increasingly contributing to the Irish economy during the study period. Therefore, population unemployment was considered as an additional SES indicator in discrete and composite sensitivity analyses (Sensitivity Analysis 2).

Although an SES indicator capturing foreign nationality was utilised in discrete and composite analyses for consistency with previous social research [16], [48]–[50], the population represented by the non-Irish/non-UK nationality indicator was extremely diverse. For example, non-Irish/non-UK nationals were typically younger, with higher educational statuses, and greater labour force participation rates than their Irish/UK counterparts; however, non-Irish/non-UK nationals were also more likely to be working in manual occupations with a frequency of unskilled workers approximately twice that of Irish/UK nationals [51]. Therefore, since the foreign nationality indicator may not have been a clear measure of deprivation in the Irish context, an additional composite sensitivity analysis (Sensitivity Analysis 3) was conducted with the exclusion of the non-Irish/non-UK nationality variable, also substituting population unemployment for male unemployment due to the clearer trends identified in prior discrete analyses.

After examining post-ban effects by both discrete SES indicators and composite SES indices, sensitivity analyses were conducted to test post-ban effects by the structural and material factors that were generated and extracted during prior principal component analyses. Each of the separate factors was assigned to mortality events by local authority areas, and the distribution was divided into tertiles for the subsequent interrupted time-series Poisson regression analysis. These sensitivity analyses were conducted with the separate factors for both the baseline index and Sensitivity Index 3, which was identified as the most appropriate composite index based upon the percentage variance of the individual variables explained by the two factors.

## Results

Table 1 displays the descriptive statistics, expressed as percentages, for each of the SES indicators representing conditions of deprivation across the 34 local authority areas. The Spearman correlation coefficients highlighted the complex relationships between SES indicators (Table 2). For example, foreign status as a non-Irish/non-UK national was inversely correlated with all indicators except for a weakly positive correlation with population unemployment (0.10) and a moderately positive correlation with rented/free housing tenure (0.41). In turn, rented/free housing tenure was positively correlated with both male (0.56) and population unemployment (0.61) as well as with having no car access (0.60).

**Table 1:** Descriptive Statistics of Area-Level Socioeconomic Indicators, Republic of Ireland, 2000–2010.

Socioeconomic Indicators	Mean (S.D.) (%)	Median Value (%)	Coefficient of Variation (%)	1^st^ and 2^nd^ Tertile Cutoff Value (%)	2^nd^ and 3^rd^ Tertile Cutoff Value (%)
Low Education	24.3 (5.3)	24.3	21.8	22.0	26.6
Manual Occupation	35.9 (4.9)	36.6	13.6	34.7	38.0
Non-Irish/Non-UK Nationality	5.8 (3.4)	5.2	58.6	3.9	6.6
≥5 Person Families	18.3 (4.2)	18.3	22.9	16.3	20.3
Male Unemployment	5.5 (1.6)	5.2	29.1	4.7	5.8
Population Unemployment*	4.5 (1.1)	4.3	24.4	4.0	4.7
Rented/Free Housing	22.3 (7.1)	20.4	31.8	18.7	21.6
No Car Access	18.9 (7.1)	16.6	37.5	15.3	18.5

*For sensitivity analyses.

**Table 2:** Spearman Correlation Coefficient Matrix for Area-Level Socioeconomic Indicators, Republic of Ireland, 2000–2010.

Socioeconomic Indicators	Low Education	Manual Occupation	Non-Irish/UK Nationality	≥5 Person Families	Male Unemployment	Population Unemployment*	Rented/Free Housing	No Car Access
Low Education	1.00							
Manual Occupation	0.64	1.00						
Non-Irish/UK Nationality	−0.56	−0.52	1.00					
≥5 Person Families	0.58	0.42	−0.82	1.00				
Male Unemployment	0.54	0.30	−0.10	−0.02	1.00			
Population Unemployment*	0.42	0.26	0.10	−0.19	0.94	1.00		
Rented/Free Housing	−0.03	−0.25	0.41	−0.53	0.56	0.61	1.00	
No Car Access	0.63	0.17	−0.10	0.07	0.78	0.70	0.60	1.00

*For sensitivity analyses.

The baseline PCA yielded two factors explaining 81% of the overall variance. The principal component rotated matrix confirmed that the results of the sensitivity analyses were comparable to the baseline PCA in the number of factors identified for extraction and the clear division between the structural and material aspects of SES represented by the factor loadings (Table 3). The proportion of the overall variance explained by the factors was also similar across all composite indices with Sensitivity Analyses 1–3 respectively explaining 81%, 80%, and 82% of the overall variance.

**Table 3:** Principal Component Rotated Matrix for Composite Socioeconomic Indices, Republic of Ireland, 2000–2010.

	Baseline Analysis		Sensitivity Analysis 1†		Sensitivity Analysis 2‡		Sensitivity Analysis 3§	
Socioeconomic Indicators	Factor 1	Factor 2	Factor 1	Factor 2	Factor 1	Factor 2	Factor 1	Factor 2
Low Education	0.85	−	0.81	−	0.88	−	−	0.91
High Education	−	−	-0.92	−	−	−	−	−
Manual Occupation	0.75	−	0.79	−	0.77	−	−	0.83
Non-Irish/Non-UK Nationality	−0.84	−	−0.84	−	−0.81	−	−	−
≥5 Person Families	0.82	−	0.81	−	0.80	−	−	0.74
Male Unemployment	−	0.87	−	0.89	−	−	−	−
Population Unemployment	−	−	−	−	−	0.87	0.84	−
Rented/Free Housing	−	0.85	−	0.80	−	0.86	0.90	−
No Car Access	−	0.95	−	0.93	−	0.93	0.94	−

† Including High Education.

‡ Substituting Male Unemployment with Population Unemployment.

§ Substituting Male Unemployment with Population Unemployment and Excluding Nationality.

From 2000–2010, there were 99,466 total deaths due to IHD (n = 60,071), stroke (n = 24,203), and COPD (n = 15,192) in the Irish population, ages ≥35 years. Seasonal variation was observed, with the largest number of mortality events occurring in winter. Increased ILI activity was detected during eight periods, with the most extended increase occurring for approximately three months of the 2009–2010 influenza season. Smoking prevalence remained relatively stable with an absolute, unadjusted decline of 2% over the study period. Consistent with previously published analyses over a 2000–2007 study period [1], no post-ban annual trend effects were detected for any causes of death (data not shown). Therefore, only SES differentials in immediate post-ban mortality effects are reported for the remainder of the study.

**Figure 1:**
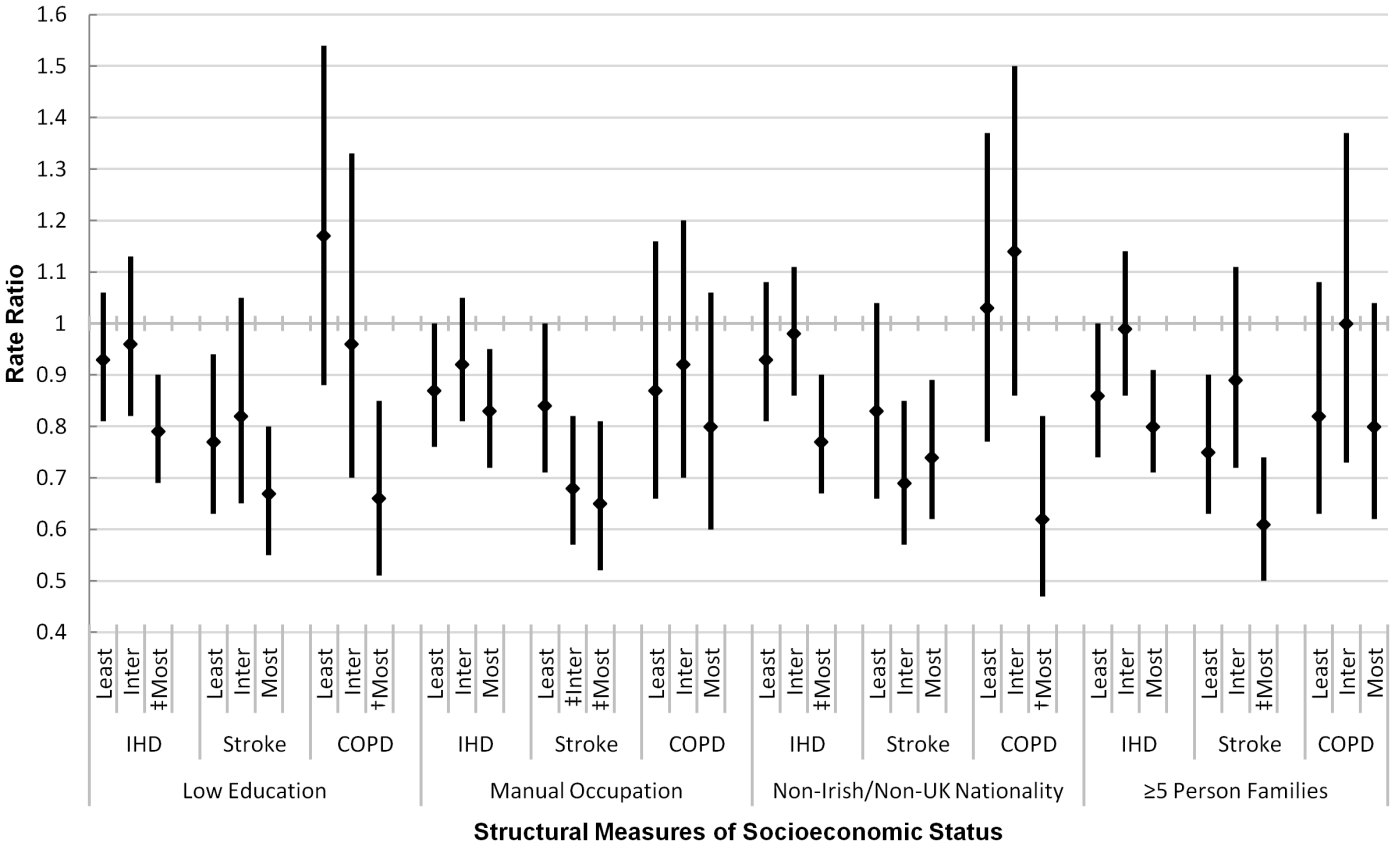
Immediate Post-Smoking Ban Effects^§^ on Cause-Specific Mortality by Structural Measures of Socioeconomic Status, Ages ≥35 Years, Republic of Ireland, 2000–2010*. ^§^Age and gender-standardised and adjusted for time trend, season, influenza, and smoking prevalence. *‘Least’ refers to the least deprived tertile, ‘Inter’ to the intermediate tertile, and ‘Most’ to the most deprived tertile IHD  =  ischemic heart disease COPD  =  chronic obstructive pulmonary disease. ^†^Significantly different from least deprived tertile at 95% confidence level. ^‡^Significantly different from least deprived tertile at 90% confidence level.

Post-ban mortality effects by structural SES indicators are shown in Figure 1. Overall, effects were concentrated in the most deprived tertile across all causes of death, indicating post-ban reductions in smoking-related inequalities. Specifically, effects by low education were exhibited only in the most deprived tertile for IHD and COPD, and in both the least and most deprived tertiles for stroke with statistically similar effects. When examined by manual occupation and families of ≥5 persons, IHD and stroke effects were strongest in the most deprived tertiles, with no effects observed for COPD. Post-ban IHD and COPD effects were only detected in local authority areas of Ireland with the greatest frequency of non-Irish/non-UK nationals, with statistically similar stroke effects detected in both the intermediate and most deprived groups.

**Figure 2:**
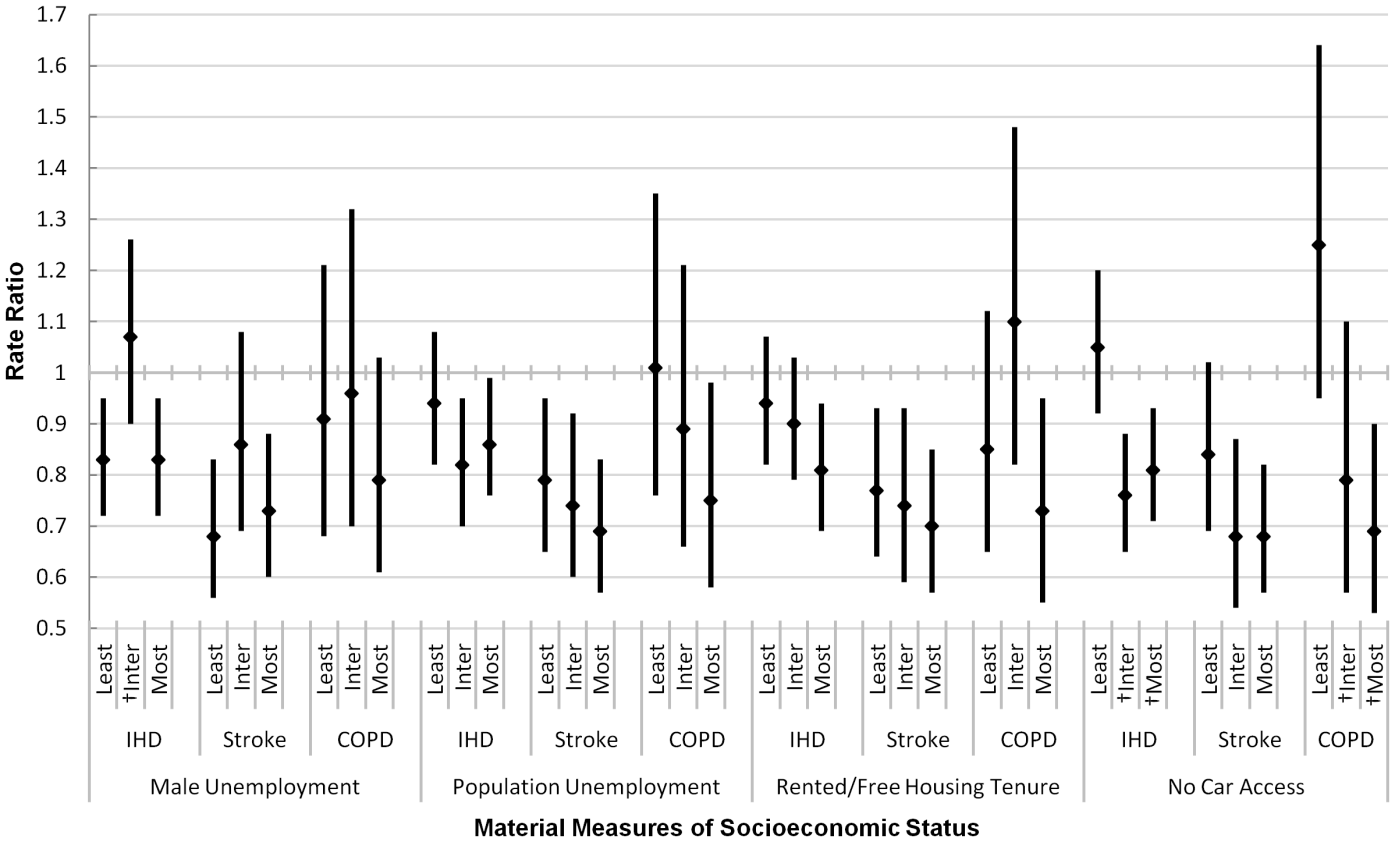
Immediate Post-Smoking Ban Effects^§^ on Cause-Specific Mortality by Material Measures of Socioeconomic Status, Ages ≥35 Years, Republic of Ireland, 2000–2010*. ^§^Age and gender-standardised and adjusted for time trend, season, influenza, and smoking prevalence. *‘Least’ refers to the least deprived tertile, ‘Inter’ to the intermediate tertile, and ‘Most’ to the most deprived tertile IHD  =  ischemic heart disease COPD  =  chronic obstructive pulmonary disease. ^†^Significantly different from least deprived tertile at 95% confidence level. ^‡^Significantly different from least deprived tertile at 90% confidence level.

Post-ban immediate mortality effects by material SES indicators are shown in Figure 2. The overall trend indicated equitable mortality reductions across SES tertiles, with statistically similar effects detected by male unemployment, population unemployment, and rented/free housing tenure. When ban effects were examined by the no car access indicator, reductions in inequalities were detected, with greater effects observed in the intermediate and most deprived tertiles as compared to the least deprived tertile. Male unemployment did not yield effects consistent with that of the other material measures. However, analyses by population unemployment yielded a clearer trend, also mirroring results by rented/free housing tenure.

**Figure 3:**
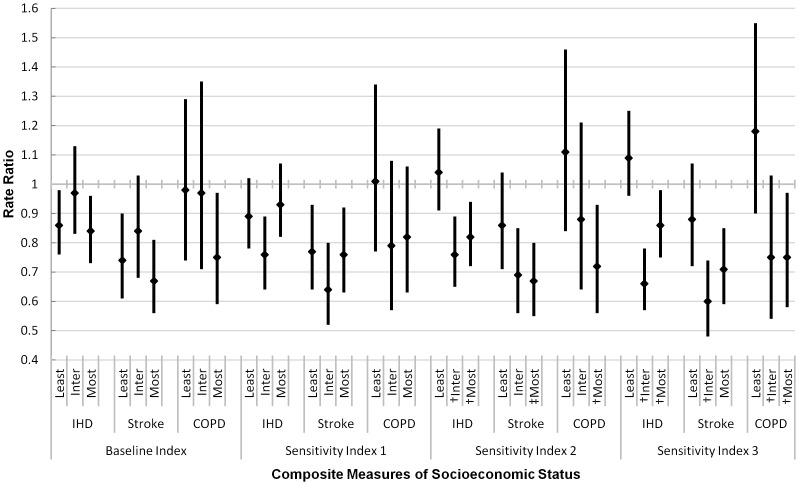
Immediate Post-Smoking Ban Effects^§^ on Cause-Specific Mortality by Composite Measures^¶^ of Socioeconomic Status, Ages ≥35 Years, Republic of Ireland, 2000–2010*. ^§^Age and gender-standardised and adjusted for time trend, season, influenza, and smoking prevalence. ^¶^Baseline Index includes Low Education, Manual Occupation, Non-Irish/Non-UK Nationality, ≥5 Person Families, Male Unemployment, Rented/Free Housing Tenure, and No Car Access. Sensitivity Index 1 includes Baseline Index and High Education. Sensitivity Index 2 substitutes Male Unemployment with Population Unemployment. Sensitivity Index 3 substitutes Male Unemployment with Population Unemployment and excludes Nationality. *‘Least’ refers to the least deprived tertile, ‘Inter’ to the intermediate tertile, and ‘Most’ to the most deprived tertile. IHD  =  ischemic heart disease COPD  =  chronic obstructive pulmonary disease. ^†^Significantly different from least deprived tertile at 95% confidence level. ^‡^Significantly different from least deprived tertile at 90% confidence level.

Post-ban effects by the baseline and sensitivity composite indices are shown in Figure 3. IHD and COPD effects were attenuated in the composite index when compared to effects by discrete SES indicators, but composite stroke effects generally fell within the confidence limits of the discrete effects. Both the baseline index and Sensitivity Analysis 1 indicated equitable mortality reductions across SES tertiles, consistent with the overall effects detected by the discrete, material SES indicators. However, the results of Sensitivity Analyses 2 and 3 demonstrated reductions in inequalities, with statistically greater effects detected in the intermediate and most deprived tertiles when compared to the least deprived tertile, closely mirroring overall effects detected by the discrete, structural SES indicators.

Immediate post-ban effects by the separate factors extracted in the principal component analyses for both the baseline index and Sensitivity Index 3 are displayed in Figure S1. Factor 1 of the baseline index, characterised by the structural SES indicators, demonstrated greater effects in the most deprived and intermediate tertiles across all causes of death. Factor 2 of the baseline index, characterised by the material SES indicators, demonstrated post-ban mortality reductions that were concentrated in both the most deprived and intermediate tertiles, which were statistically stronger for IHD and COPD, and statistically similar across SES tertiles for stroke. In contrast to the baseline index, Factor 1 of Sensitivity Index 3 was characterised by the material SES indicators, and Factor 2 was characterized by the structural SES indicators. When these factor-specific post-ban effects were compared to the overall effects for Sensitivity Index 3 (Figure 3), the material factor clearly functioned as the driver of the overall composite index, with statistically stronger effects observed in the most deprived and intermediate tertiles as compared to the least deprived tertile.

## Discussion

Overall findings indicate that in the month following the implementation of the national Irish smoking ban, inequalities in smoking-related mortality were reduced. Since the observed post-ban mortality decreases were either greater or similar in the most deprived tertile when compared to the least deprived tertile, reductions in inequalities occurred due to the existing higher rates of smoking-related mortality in the most deprived SES group. Although the choice of SES indicator influenced the measurement of effects, results were broadly consistent across discrete indicators and composite indices, demonstrating that the Irish national smoking ban did not widen inequalities and, in some cases, largely reduced inequalities in smoking-related mortality.

As this was the first study to assess post-smoking ban effects by discrete SES indicators, direct comparisons cannot be made with any other studies. However, the contextual applicability of the structural and material indicators was confirmed by the results of their combined assessment in the PCA, yielding two clearly divisible components. One factor characterised the structural aspects of SES, with high loadings on education, occupation, foreign nationality, and family composition. This is consistent with what is previously known in that education and occupation are important in determining social status and social identity [23], [41]. There is also an occupational social gradient in smoking prevalence that is consistent with the social gradient in mortality, attributable to the earlier age of beginning smoking and lower rates of cessation among lower SES groups [52]. In addition to the social gradient in smoking prevalence, evidence has also revealed a gradient in nicotine intake, with smokers of lower SES smoking more cigarettes and inhaling each cigarette more intensively than affluent smokers [52]–[54]. This higher intake results in a stronger physical addiction to nicotine, making it more difficult for those of lower SES to cease smoking even when exhibiting the psychological intent to quit [52], [53].

Furthermore, family composition and foreign nationality may also function as structural determinants of social standing. Large families, defined as families with three or more children, are associated with poverty, and resources become increasingly diluted as the number of children increases [55]. This concept becomes linked with foreign nationality through the higher fertility rates of non-European Union (EU) migrants [56], [57]. Additional data for Europe indicate that migrants from outside the EU have greater rates of unemployment when compared to EU migrants or native country citizens [41] and migrants from any country are more vulnerable to social exclusion [58], [59]. As the 2002 Irish census did not differentiate between EU and non-EU migrants, it was not possible to distinguish effects between these groups in this study.

The other factor identified through PCA characterised the material aspects of SES, with high loadings on unemployment, housing tenure, and car access. These concepts are closely associated in that unemployed persons are more likely to lack material resources, to live in rented housing, and to be without car access when compared to their employed counterparts [60]. Job insecurity is also associated with cardiovascular disease and with the risk factors for cardiovascular disease [61], which can result in increased risk of mortality. Further to this, persons living in rented housing and persons without car access have higher mortality rates when compared to house owner-occupiers and car owners [62]. Potential explanations are that living in badly maintained rented housing can result in exposures to environmental risk factors, such as pollution and mould, and psychological risk factors, such as the questionable safety of physical surroundings, while the lack of car access may decrease employability, access to health services, and engagement with social support networks [62]. Consequently, smoking is heavily employed as a coping mechanism for these stressors [42], [52], [63], resulting in increased population exposure to secondhand smoke in social and workplace settings. For the most deprived groups, secondhand smoke exposure acts concurrently with these other disadvantaged circumstances to yield an increased risk of negative health outcomes. Thus, the mortality benefits experienced by the most deprived in Ireland indicate that the implementation of the national Irish smoking ban was effective in immediately reducing this harmful exposure to secondhand smoke.

When compared to effects by discrete SES indicators, the composite index yielded attenuated effects for IHD and COPD, but effectively captured the magnitude of discrete SES effects for stroke. This finding implies that SES indicators may not always measure inequalities similarly across causes of death. A potential explanation is that IHD, stroke, and COPD are distributed differently across demographic groups. For instance, IHD is responsible for more premature deaths in persons ≤65 years than COPD, which disproportionately affects persons ≥65 years. This results in different risk factor distributions that are closely associated with SES indicators. Additionally, the mechanisms by which secondhand smoke exposure can trigger biological responses are disease-specific and may, therefore, result in different effects when the exposure is reduced or removed. For example, exposure to secondhand smoke can result in endothelial dysfunction, leading to ischemic heart disease and increased risk of mortality for those with existing disease; however, the endothelial repair mechanism partially recovers when the exposure is removed, partially accounting for the decreases in ischemic heart disease mortality following smoking ban implementation [64], [65]. Though secondhand smoke exposure has been causally linked to ischemic heart disease, limited evidence exists for establishing a causal association between secondhand smoke exposure and stroke or COPD; thus, the evidence is currently classified as suggestive [66]–[70]. As a result, these disease-specific biological response mechanisms have not yet been fully elucidated and present a generative area for further research exploration.

Although Sensitivity Analysis 1 resulted in similar factor loadings to the baseline PCA, the inclusion of the high education variable did not increase the explanatory power for the overall variance and the resulting composite index did not show clear trends in mortality effects. As such, the high education variable did not serve as an appropriate predictor of health inequalities in the Irish context. However, the composite index arising from Sensitivity Analysis 2, substituting population unemployment for male unemployment, provided a clearer trend and coincided more closely with the discrete SES analyses than the baseline PCA. As such, population unemployment was retained in Sensitivity Analysis 3, which also excluded the indicator for foreign nationality, resulting in the most appropriate composite index that accounted for the most overall variance.

These additional analyses demonstrated that the construction of the composite index was quite sensitive to the variables included. Nevertheless, the composite index generated through PCA was likely the best measure for identifying SES effects, inherently accounting for both the structural and material aspects of SES. However, discrete analyses were a useful first step in understanding how individual indicators served as measures of health inequalities and in providing critical information regarding the most appropriate indicators to include in the composite index. Such a differentiated, analytical approach aided in assessing the validity of the overall estimation of SES effects.

The findings from the sensitivity analyses conducted with the separate factors generated through PCA were consistent with the overall effects resulting from stratification by the discrete structural and material SES indicators. Factor 1 of Sensitivity Index 3, the material factor, was likely the best factor measure of SES in this study, as it was responsible for explaining most of the overall variance of the individual variables, driving the effects demonstrated by the composite index most appropriate to the study population (Sensitivity Index 3). Consistent with previous findings by the discrete and composite SES measures, reductions in inequalities were observed in analyses by each of the separate factor measures.

Only two epidemiological studies of smoking ban effects in other countries have examined post-ban mortality differentials by SES measures in an adult population. One study examined rates of acute coronary events, including hospital admissions and out-of-hospital deaths, in the city-wide population of Rome, Italy [16]. In ages 35–64 years, post-ban reductions were observed in the three lowest SES quintiles, with the largest reductions occurring in the lowest SES quintile, whereas in ages 65–74 years, effects were observed only in the second lowest SES quintile [16]. Another study examined stroke effects, including hospital admissions and out-of-hospital deaths, in the national population of Scotland, demonstrating that stroke reductions occurred only in ages <60 years and only in the two highest SES quintiles [15]. Although a third epidemiological study examined the post-ban SES effect differentials of asthma hospital admissions and deaths in Scotland, the study population was composed of children ≤14 years of age and only five deaths were identified over the study period of 9.75 years [14]; therefore, mortality differentials could not be accurately deduced. Nonetheless, direct comparability of findings from any of the above studies is not possible due to their inclusion of hospital admissions in the estimation of post-ban effects and due to the differing definitions and distributions of SES indicators in Italy, Scotland, and Ireland.

Overall evidence of smoking ban policy impacts on health inequalities is extremely limited. Only two other studies have assessed the health effects of smoking ban policies by SES. A study conducted in Christchurch, New Zealand, assessed the effects of the national smoking ban on hospital admissions due to acute myocardial infarction and found that post-ban effects were only observed for ages 55–74 years in the second highest SES quintile [13]. The other study assessed the effects of the national English smoking ban on hospital admissions for childhood asthma in ages ≤14 years and findings indicated that post-ban childhood asthma effects were similar across all SES quintiles [17]. Since only a handful of studies have examined post-ban differentials by SES and have measured different health outcomes in various cultural contexts, the findings are challenging to generalise. However, this study of the effects of the national Irish smoking ban contributes evidence to indicate that smoking ban policies are associated with reductions in inequalities in smoking-related mortality.

There are two potential mechanisms, likely acting in concurrence, to explain why the observed immediate mortality reductions have generally resulted in greater benefits for the more disadvantaged population. First, smoking is socially distributed, with a greater prevalence in the more disadvantaged groups, thus resulting in a greater risk of exposure to secondhand smoke [71], [72]. Second, there is also a greater prevalence of non-communicable diseases in the more disadvantaged groups, particularly in developed countries [7], resulting in a larger at-risk population in which exposure to secondhand smoke could trigger a negative health outcome. These risks were immediately reduced when smoking was banned in workplaces, pubs, and other social environments, plausibly resulting in greater effects for the most disadvantaged groups. The findings of previous analyses provided confirmatory evidence showing that the immediate post-ban mortality reductions were largely due to reductions in exposure to secondhand smoke [1]. The explanations for both of these mechanisms reinforce the fundamental principles for population prevention strategies wherein a shift in the exposure distribution acting on a large at-risk population produces substantial public health benefits [6].

As with all routine mortality data, information was not available on individual risk factors such as body mass index, physical activity level, and smoking status; hence, it was not possible to adjust for these in analyses. However, the most current information from the national Survey of Lifestyle, Attitudes, and Nutrition (SLÁN) in Ireland demonstrated that obesity prevalence and physical activity levels remained stable across the 1998, 2002, and 2007 survey waves [73]. All regression models included adjustments for population smoking prevalence. Additionally, previous evidence has shown that cigarette price increases, health warnings on cigarette packaging, and advertising bans in Ireland were not sufficient to explain the large, immediate mortality reductions occurring after implementation of the national workplace smoking ban [74]. Levels of enforcement can influence the effectiveness of smoking ban policies in yielding health benefits; however, compliance with the national Irish workplace smoking ban was strong (94%) immediately following policy implementation and remained strong over the entire study period [75].

SES indices were limited to local authority areas, geographic classifications wherein heterogeneity in SES indicators may exist. However, for Ireland the local authority was the smallest area-level classification available within the de-identified mortality data. Likewise, other epidemiologic studies have used the area-level of local authority for analyses of health-related outcomes [19], [76] and previous research has indicated that the choice of geographical classification, whether at the level of neighbourhood, post code sector, or borough, does not appreciably impact the size of health differences by area deprivation [43]. Furthermore, the characteristics of an area can provide the context of conditions that influence individual health risks [22].

Strengths of this study include analyses over the longest post-ban period to date, 6.75 years, and further validation of previously reported immediate effects following the implementation of the national Irish workplace smoking ban [1]. This study was unique in examining the influence of discrete SES indicators on post-ban effect differences in a national population and in providing evidence of SES effect differences in COPD mortality, which has not been reported in any previous studies. In addition, this study contributed to the sparse evidence currently available regarding the SES differences in post-ban IHD and stroke effects, now demonstrating that smoking ban policies do not widen health inequalities and, in some cases, may even reduce them. The Ireland-specific composite SES index generated through PCA was based upon the most relevant census data for the study period, and composite analyses provided corroborative evidence to discrete SES results. The findings of this study have demonstrated the immense public health impacts of smoking ban policies.

## Conclusion

Overall findings suggest that in the month following the implementation of the national Irish smoking ban, inequalities in smoking-related mortality were reduced. For IHD and COPD, mortality decreases were generally detected either solely or most strongly in the most deprived tertile, while decreases in stroke mortality were generally observed more equitably across SES groups. Regardless, the higher rates of smoking-related mortality in the most deprived group indicate that even equitable reductions across SES tertiles result in decreases in inequalities. The choice of SES indicator was influential in the measurement of effects, underscoring that a differentiated analytical approach is useful for understanding the complexities in which structural and material factors influence mortality.

## Supporting Information

Figure S1 Immediate Post-Smoking Ban Effects^§^ on Cause-Specific Mortality by Separate Factor Measures^¶^ of Socioeconomic Status, Ages ≥35 Years, Republic of Ireland, 2000–2010*. ^§^Age and gender-standardised and adjusted for time trend, season, influenza, and smoking prevalence. ^¶^Factor 1 of the Baseline Index loaded highly on the structural SES indicators, Factor 2 of the Baseline Index loaded highly on the material SES indicators, Factor 1 of Sensitivity Index 3 loaded highly on the material SES indicators, and Factor 2 of Sensitivity Index 3 loaded highly on the structural SES indicators. *‘Least’ refers to the least deprived tertile, ‘Inter’ to the intermediate tertile, and ‘Most’ to the most deprived tertile IHD  =  ischemic heart disease COPD  =  chronic obstructive pulmonary disease. ^†^Significantly different from least deprived tertile at 95% confidence level. ^‡^Significantly different from least deprived tertile at 90% confidence level.
(TIF)
