# Supplementary figures and images for: Socioeconomic Differentials in the Immediate Mortality Effects of the National Irish Smoking Ban
Source: PLoS One. 2014 Jun 2;9(6):e98617. doi: 10.1371/journal.pone.0098617 (PMC4041857; doi:10.1371/journal.pone.0098617)

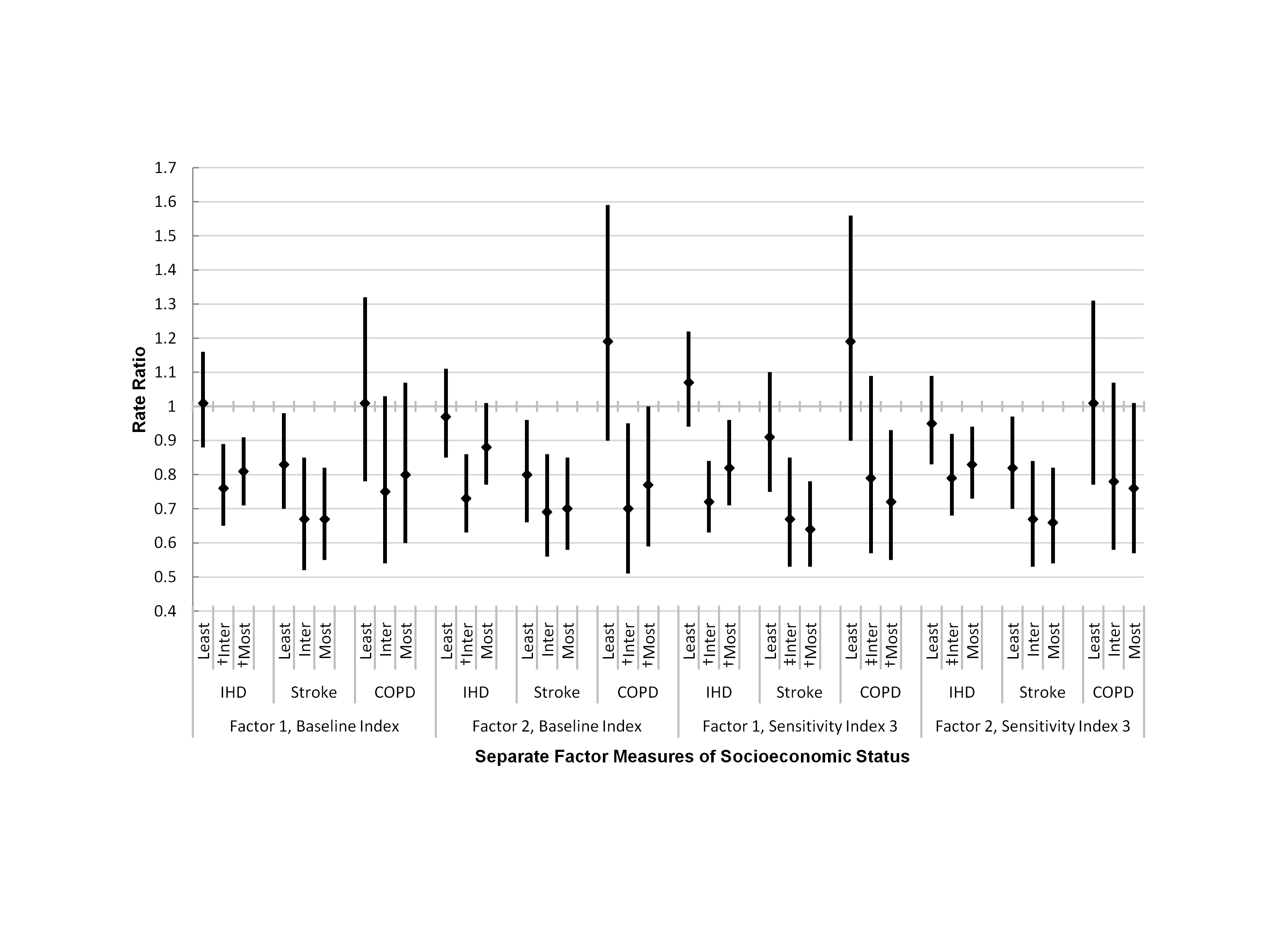

Supplement: Figure S1 — Immediate Post-Smoking Ban Effects§ on Cause-Specific Mortality by Separate Factor Measures¶ of Socioeconomic Status, Ages ≥35 Years, Republic of Ireland, 2000–2010*. §Age and gender-standardised and adjusted for time trend, season, influenza, and smoking prevalence. ¶Factor 1 of the Baseline Index loaded highly on the structural SES indicators, Factor 2 of the Baseline Index loaded highly on the material SES indicators, Factor 1 of Sensitivity Index 3 loaded highly on the material SES indicators, and Factor 2 of Sensitivity Index 3 loaded highly on the structural SES indicators. *‘Least’ refers to the least deprived tertile, ‘Inter’ to the intermediate tertile, and ‘Most’ to the most deprived tertile IHD = ischemic heart disease COPD = chronic obstructive pulmonary disease. †Significantly different from least deprived tertile at 95% confidence level. ‡Significantly different from least deprived tertile at 90% confidence level. (TIF) [file pone.0098617.s001.tif]
